# Inhaled Antibiotics in Non-cystic Fibrosis Bronchiectasis (NCFB): A Systematic Review of Efficacy and Limitations in Adult Patients

**DOI:** 10.7759/cureus.30660

**Published:** 2022-10-25

**Authors:** Zainab Amjad, Abdelrahman Abaza, Advait M Vasavada, Akhil Sadhu, Carla Valencia, Hameeda Fatima, Ijeoma Nwankwo, Mahvish Anam, Shrinkhala Maharjan, Sai Sri Penumetcha

**Affiliations:** 1 Internal Medicine, California Institute of Behavioral Neurosciences & Psychology, Fairfield, USA; 2 Internal Medicine, King Faisal University, Al Ahsa, SAU; 3 Pathology, California Institute of Behavioral Neurosciences & Psychology, Fairfield, USA; 4 Family Medicine, California Institute of Behavioral Neurosciences & Psychology, Fairfield, USA; 5 Research, California Institute of Behavioral Neurosciences & Psychology, Fairfield, USA; 6 General Medicine, California Institute of Behavioral Neurosciences & Psychology, Fairfield, USA; 7 General Medicine, Chalmeda Anand Rao Institute of Medical Sciences, Karimnagar, IND

**Keywords:** inhaled antibiotics, non-cystic fibrosis bronchiectasis, prevention of infections, eradication therapy, potency, side effects, safety and efficacy

## Abstract

Non-cystic fibrosis bronchiectasis has recently been under the spotlight due to a better detection rate with advanced imaging techniques. Recurrent infections in such patients are the main cause of their deterioration. This invariably leads to a catastrophic wheel of decline in lung function, reinfection, and repeated hospital consultations. The main goal of their management is based on the principles of prevention and vigorous treatment of recurrent infections. This review aimed to gather recent therapeutic options for inhaled antibacterial use in such patients and compare them for their properties of safety and efficacy.

Studies done in the last 10 years on adult patients were gathered using the Medical Subject Headings (MeSH) strategy and later sorted using the inclusion/exclusion criteria. Research engines used include Google Scholar, PubMed, and the Saudi Digital Library. Out of the 31,739 articles identified initially, 1362 were screened. The final eight selected papers were assessed for quality by using the quality assessment checklist, the Cochrane bias assessment tool, the Scale for the Assessment of Narrative Review Articles (SANRA) tools and cross-examined by co-authors.

We concluded that the use of inhaled antibiotics as an adjuvant and follow-up treatment option is associated with better short and long-term prognoses in patients. They lead to lesser systemic side effects than the oral and intravenous varieties available on the market. However, the establishment of a hierarchy among the subgroups remains a grey area that needs further research.

## Introduction and background

Bronchiectasis refers to a heterogeneous group of respiratory diseases that are characterized by permanent abnormal dilation of the bronchi that leads to chronic productive cough, recurrent infections, and dyspnea [1,2]. It is classified into two broad categories: cystic fibrosis (CF) and non-cystic fibrosis (NCF), based on etiology. CF is a multisystem disease inherited as an autonomous recessive disorder and presents at an early age. This genetic defect causes an increase in sodium and chloride content in sweat and increased resorption of sodium and water from the respiratory epithelium, thus leading to a dehydrated and exposed airway. Among those of the NCF variety, there are many subtypes that have a similar presentation and can be diagnosed by history, relevant examination, and thorough investigations [1]. These subtypes include congenital defects affecting the ciliary function in the respiratory epithelium, inherited disorders of immunity, destructive infections, inhalation of toxins, and foreign body aspiration. Failure to identify the underlying cause is a frequent concern in many patients. In recent years, an increase in incidence has been reported in the NCF variety due to physicians’ awareness and the easy availability of radiological imaging [2]. Chest x-ray and high-resolution computerized tomography scan are the imaging modalities of choice in such patients. Radiological findings most consistently found include thickened and dilated airways that are most markedly found in the peripheries of the lung parenchyma [3].

An increase in mortality and morbidity secondary to declining lung function tests has been associated with the presence of microbes in the airways [4]. The most commonly isolated pathogen is *Pseudomonas aeruginosa* (PA), followed by *Haemophilus influenzae*, *Streptococcus*, and *Staphylococcus* [5]. *Non-tuberculous mycobacterium*, viral and fungal infections are also less frequently responsible for exacerbations. Irreversible structural remodeling of the airways leads to a vicious cycle of infections which further propels the person toward progressive loss of lung function [6]. Acute and preventive management of infections is therefore pertinent to reducing bronchial inflammation, improving quality of life, and achieving long-term positive outcomes [7,8]. Inhaled antibiotics are generally considered more effective and safer than oral and intravenous varieties. However, some experts differ in their opinion [9,10]. They argue that although such interventions reduce the number of exacerbations, sputum production, and bacterial load in the airways, they also cause more localized side effects that can result in their withdrawal. The side effects most commonly reported include cough, bronchospasm, and acute exacerbation.

Comparative trials on inhaled antibiotics are limited and controversial due to the bias in population selection and methodological variability. Researchers also suggest that treatment options used for the management of CF varieties should not be replicated in NCF patients due to a lack of evidence and contradictions in benefits [11]. There is no known cure for bronchiectasis except lung transplantation, which is currently being offered only to end-stage patients [12]. This systematic review aims to scrutinize the various forms of inhaled antibiotics used for the treatment of NCF in the adult population. This will help further to identify the options that cause the least side effects while executing similar results as their counterparts.

## Review

Methods

Using the preferred reporting items for systematic reviews and meta-analysis (PRISMA) guidelines [13], after the selection of the final topic and identification of keywords, the Medical Subject Headings (MeSH) strategy was obtained and later used for the generation of articles in PubMed. The final MeSH strategy used for PubMed, PMC is as follows: Safety and efficacy OR side effects OR adverse effects OR effectiveness OR potency OR value OR eradication OR prevention OR treatment OR "Clinical Trials Data Monitoring Committees"(Majr) AND Bronchiectasis OR permanent airway dilation OR ("Bronchiectasis/drug therapy"(Majr) OR "Bronchiectasis/microbiology"(Majr) OR "Bronchiectasis/mortality"(Majr) OR "Bronchiectasis/prevention and control"(Majr) OR "Bronchiectasis/therapy"(Majr)) AND Antibiotic OR anti-microbial therapy OR anti-bacterial medication ("Anti-Bacterial Agents/adverse effects"(Majr) OR "Anti-Bacterial Agents/drug therapy"(Majr) OR "Anti-Bacterial Agents/microbiology"(Majr) OR "Anti-Bacterial Agents/pharmacokinetics"(Majr) OR "Anti-Bacterial Agents/therapeutic use"(Majr) OR "Anti-Bacterial Agents/therapy"(Majr) OR "Anti-Bacterial Agents/toxicity"(Majr)).

**Figure 1:**
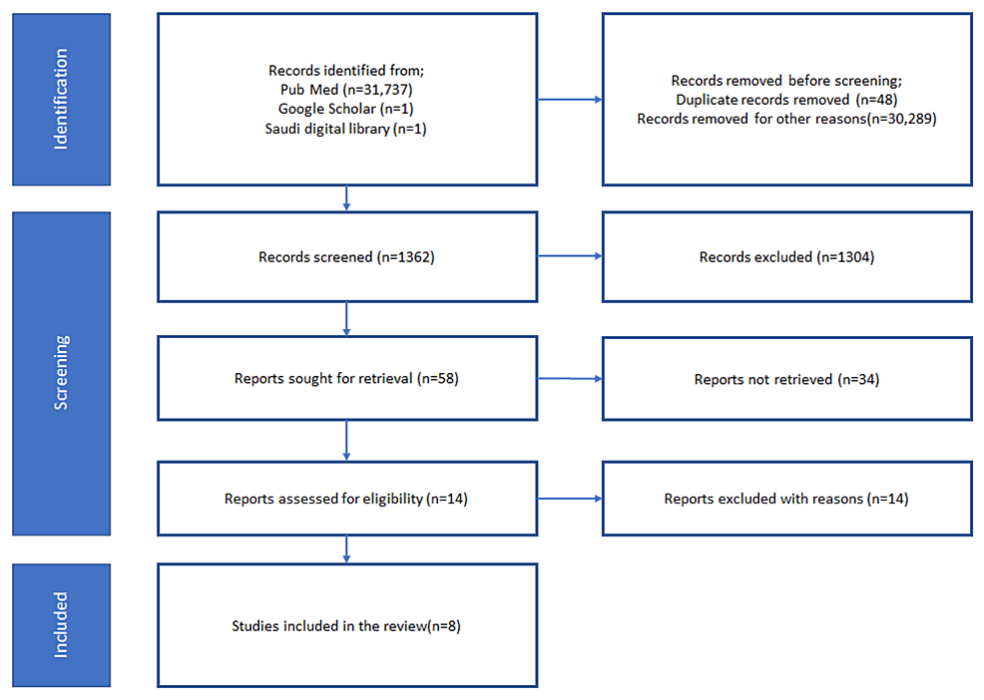
Literature review search flowsheet using PRISMA 2020 guidelines. n: number; PRISMA: preferred reporting items for systematic reviews and meta-analysis.

Later, the inclusion and exclusion criteria were added, and the research was refined. All articles more than 10 years old, based on species other than human species, in a language other than English, published in books and documents, and simple review studies were excluded. All other articles based on the adult population were included. Following the identification of relevant articles, duplicates were screened and removed. A manual search of articles was also done on Google Scholar and the Saudi Digital Library. In the next phase, all articles identified were screened by their abstracts, and retrieved articles fulfilling requirements were selected. In the third phase, the availability of free full text was assessed and this led to the final list that was scrutinized for eligibility. All eligible articles were then evaluated with a quality assessment checklist, the Cochrane bias assessment tool, and the Scale for the Assessment of Narrative Review Articles (SANRA) checklist to ensure their validity. The search strategy and quality appraisal were performed independently by two different authors. Figure 1 illustrates the search strategy used to follow the principles of the PRISMA guidelines [13].

Results

A total of 31,743 articles were identified, out of which 48 duplicate records were detected. After modifying the search using inclusion and exclusion criteria, another 30,289 articles were removed. One thousand three hundred sixty-two articles were screened by title and abstract, which led to the removal of 1304 articles and the retention of the rest. The 58 articles generated were reduced to 14 after checking for relatability. Fourteen of the final selected articles were assessed for the availability of free full text and validity using the tools mentioned previously. Eight articles were finalized and used for the write-up of the systemic review. A breakup of the articles includes six randomized controlled trials (RCTs) and one prospective and one historic cohort. Table 1 summarizes the key points, outcomes, and statistics of the selected articles.

**Table 1:** Summary of the key points, outcomes, and statistics of the selected articles. DPI: dry powder inhaler; RCT: randomized controlled trial; LFT: lung function test; SC: sputum culture; QOLS: quality of life scale; SE: side effects; NOA: number of admissions; NOE: number of exacerbations; BID: twice daily.

Author of the publication	Intervention	Type/year of the study	Study population	Duration of antibiotic	Monitoring parameters	Efficacy and outcome	Safety concerns
Martínez-García et al. [14]	Patients receiving at least one dose of DPI antibiotic (colistin, tobramycin)	Historic cohort/2020	164	1 day-30 month	Symptoms: LFT, sputum analysis, NOA, and NOE	Microbiological analysis showed a reduction in chronic bronchial infection, sputum production and purulence, and a decrease in admissions and emergency visits. Lung function tests and symptoms remained unaffected.	About 24.4% of withdrawals are mainly due to cough. Observed more in the tobramycin group.
Blanco-Aparicio et al. [15]	Oral ciprofloxacin 750 mg BID for three weeks or IV tobramycin 5 mg/kg plus beta-lactam antipseudomonal antibiotics for two weeks followed by self-administration of inhaled colistin (Promixin 1 MIU BID) administered with the I-neb® adaptive aerosol delivery device (Philips Respironics, Chichester, UK)	Prospective trial/2019	67	12-months	SC, blood tests (hematology, biochemistry), LFT, symptoms, NOA, emergency visits, cycles of antibiotics used, and NOE.	The eradication of Pseudomonas was not statistically significant. The NOA and antibiotic use were reduced. The incidence of a visit to the emergency room remained unaffected.	No discontinuation was reported.
Ailiyaer et al. [16]	Nebulized amikacin sulfate is administered BID by the jet atomizer. Amikacin sulfate (2 mL of 0.2 g/mL) with 3 ml of normal saline as an adjuvant to intravenous antibiotic therapy	Randomized prospective trial/2018	152	14-Days	Sputum weight, SC, and score. LFT, QOLS, and dyspnea scale.	Microbiological analysis of sputum showed drastic improvement in the intervention group. Other parameters showed no significant change.	Drug resistance was reported in five patients versus seven in the control. About 4% experienced bronchospasm. No discontinuation was reported.
Aksamit et al. [17]	Ciprofloxacin DPI 32.5 mg twice BID. Follow-up was done for eight weeks post-treatment. A total of 48-week follow-up was done.	RCT/2018	521	Fourteen days on/off (12 cycles) and ciprofloxacin DPI 32.5 mg twice daily for 28 days on/off (six cycles)	Time to the first exacerbation, NOE, worsening of signs and symptoms, QOLS, microbiological assessment, and LFT.	Both regimes increase the time to first exacerbation but are not statistically significant. Other outcomes are also not conclusive.	Overall, serious treatment-emergent SE stood at 22%. Atrial flutter was reported in one patient; nine treatment-emergent deaths were reported but not drug-related. Discontinuation due to SE was 5.7% and 3.5% in the 14-day and 28-day categories, respectively.
Orriols et al. [18]	For three months, followed by a short course of intravenous antibiotics, 300 mg of nebulized tobramycin (Tobi®, Novartis AG, Basel, Switzerland) BID. A twelve-month follow-up was done without treatment.	RCT/2015	35	Three months	SC, NOE, NOA, duration of admission, duration of antibiotic use, change in inflammatory markers, leukocyte count, LFT, and blood gases.	Overall improvement in most of the parameters in the tobramycin group.	Incidence of drug-resistant or opportunistic infections was not reported. Five patients were discontinued from the treatment group due to bronchospasm.
De Soyza et al. [19]	BID ciprofloxacin DPI 32.5 mg. Follow-up was done for eight weeks post-treatment. Total of 48-week follow-up.	RCT/2017	416	Fourteen days on/14 days off therapy: 12 active cycles over 48 weeks. Twenty-eight days on/28 days off therapy: six active cycles over 48 weeks.	Time to first exacerbation, NOE, worsening of signs and symptoms, QOLS, microbiological assessment, and LFT.	More significant outcomes were observed in the 14-day regimen versus 28 days.	The rates of discontinuation, SE, and treatment-emergent respiratory events were similar across all groups. Six treatment-emergent deaths were reported but not drug-related.
Serisier et al. [20]	Liposomal ciprofloxacin for inhalation (150 mg in 3 ml) and free ciprofloxacin (60 mg in 3 ml). Matched placebo consisted of control liposomes (15 mg in 3 ml) and normal saline (0.9%, 3 ml). Follow-up was done for 24 weeks	RCT/2013	42	Twenty-eight days of inhaled treatment	LFT, six-minute walk test, sputum collection, chest radiograph, respiratory questionnaire for symptoms and signs	Benefits are observed mostly in the delay in exacerbations and in the reduction in the bacterial load. Other parameters were not statistically significant	Well tolerated, no reported treatment-related serious SE. Treatment was discontinued in 15 patients mostly secondary to respiratory-related effects.
Wilson et al. [21]	Ciprofloxacin DPI 32.5 mg (50 mg dry powder) or matching placebo BID, dispensed from a T-326 inhaler (Novartis)’ follow-up is done for up to eight weeks.	RCT/2012	124	28 days	Bacterial density in sputum, inflammatory markers, LFT, sputum quantity and color, NOA, and the NOE.	Bacterial density and the emergence of new pathogens in sputum were reduced. In the long term, benefits were not observed.	About 31.7% withdrew from treatment due to SE. No significant bronchospasm was reported.

Discussion

Safety Profile and Concerns

The inhaled form of antibiotic serves as an alternate, cheaper mode of delivery of life-saving medication without the emergence of systemic complications. Chronic and high-dose antibacterial drugs, especially macrolides and quinolones, are associated with ototoxicity and nephrotoxicity, which can lead to failure of completion. Despite the disorder in taste that many patients who use inhaled antibacterials experience, they are still well tolerated [7]. Other common side effects reported are cough, bronchospasm, acute exacerbation, headache, and nausea. Bronchospasm and cough are the leading causes of failure of compliance among patients. Small volume atomizers (SMAs), pressured meter dose inhalers (PMDIs), and dry powder inhalers (DPIs) offer different mechanisms of delivery of inhalation drugs. It is worth mentioning that the efficacy and safety profile of a drug can vary depending on the form of atomizer being used. In order to make a better understanding of the available treatment options, we made a comparison of the drugs in accordance to the type of inhalation form used [14-21].

We were able to identify three types of inhaled non-dry antibiotics that have been researched in non-cystic fibrosis bronchiectasis (NCFB) patients. They include tobramycin, colistin, and amikacin. Colistin belongs to the polymixin class of anti-bacterials, whereas the other two are from the aminoglycoside category. In the Orriols et al. study, the use of tobramycin was associated with a 14.2% withdrawal due to bronchospasm. However, this was predominantly reported among patients having poor lung functions (median forced vital capacity (FVC) = 57.7% of predicted and forced expiratory volume in the first second (FEV1) = 34.8% of predicted) [18]. Among the patients using colistin, Blanco-Aparicio et al. observed that 7.5% of patients demonstrated mild adverse effects of cough accompanied with or without wheezing, but the medication was not discontinued [15]. In contrast, patients using nebulized amikacin in Ailiyaer et al.'s prospective study, only 4% reported bronchospasm but there was no withdrawal of treatment [16]. It is important to consider that this was a short-duration study as compared to the previously mentioned.

Recently, there has been a focus on the DPI variety of antibiotics as a form of delivery. A multicenter retrospective cohort in Spain of 164 patients in a Martínez-García et al. study included 86% of adult patients on dry powder colistin and 14% on dry powder tobramycin category [14]. In 24.4% of the total patients, treatment had to be stopped due to unwanted side effects, among which cough was the most common concern in the majority. Colistin was better tolerated, easier to use, and reported a lesser incidence of drug-resistant *Pseudomonas aeruginosa *(PA) infection. Lack of proper technique, coexisting chronic obstructive lung disease, and the presence of cough before the treatment was found to have a direct correlation to the withdrawal. Other side effects observed were dyspnea (26.8%), discomfort in the chest (15.8%), bad taste (6.1%), and hemoptysis (3.6%). A phase 2 multicenter RCT conducted by Wilson et al. compared the DPI form of ciprofloxacin, which belongs to the fluoroquinolone class of antibiotics, with a placebo for eight weeks [21]. It was noted that 31.7% of patients in the ciprofloxacin DPI category prematurely discontinued the treatment due to side effects, although those having adverse effects of significance, including bronchospasm, cough, and hemoptysis, were statistically non-significant. The respire trials are probably the most comprehensive trials done in this category. They are multinational, multicenter, randomized, double-blind prospective studies conducted in 38 countries. In respire 1, De Soyza et al. observed that 83% of patients experienced an adverse effect, among which the majority had them secondary to respiratory-related category (47.1%), followed by infections (36.8%) and gastrointestinal disorders (30%) [19]. Nervous system and general disorders both stood at 24%, whereas 20% had musculoskeletal-related adverse effects. It is worthwhile to mention that serious side effects were reported in only 1.5% of patients. It concluded that patients on ciprofloxacin DPI have a similar incidence of treatment-emergent (TE) and drug-related TE as placebo and are well tolerated by the patients. Aksamit et al., in respire 2, reported an overall 65% treatment-related SE. However, the incidence reported was lower in the longer cycle duration [17]. Respiratory-related symptoms and infections remained the most dominant category within it as well. Overall, the study’s drug-related TE SE were significantly lower in respire 2 than in 1, 13.7%, and 25.1%, respectively. One patient in the 28-day cycle sub-group suffered atrial flutter secondary to the treatment in a patient with a history of ischemic heart disease. The difference in geographical region and ethnicity may have been attributed to the overall findings of the trials. Patients in whom the trial had to be discontinued was only 5%.

Liposomal encapsulated forms of antibiotics are yet another type of antibiotic delivery mechanism. This form of ciprofloxacin has shown superiority over regular forms in terms of longer half-life and sustained higher concentrations in sputum. The orbit 2 trial was a multicenter RCT that was undertaken at multiple sites across Australia and New Zealand [20]. It demonstrated good tolerability and fewer pulmonary-related side effects, though the overall incidence was comparable to the placebo group as reported by Serisier et al.

Efficacy and Use

In the Orriols et al. study, there was a significant reduction in the number of exacerbations, hospital admission rate and duration, and use of antibiotics [18]. However, there was not much improvement in the laboratory and quality of life index monitoring scales. About 90.9% of patients free of PA were reported in the treatment group at the end of the first month and 54.5% at the end of the study. These values were 76.5% and 29.4% in the placebo group, respectively. The Blanco-Aparicio et al. study was focused on patients with NCFB having a chronic infection [15]. It demonstrated a lower eradication rate of the bacteria while using colistin, where after three months, 61.2% of patients had negative sputum cultures and 50% at 12 months. The Ailiyaer et al. study focused on short-duration adjuvant amikacin nebulizer therapy in patients presenting with exacerbation [16]. It reported an eradication rate of 51.4% in the intervention group compared to 23.2% in the control but no other improvement. The authors observed no complaints of distaste and good stability of the drug, in addition to its low cost.

The Martínez-García et al. study concluded there was no difference in endpoints between colistin and tobramycin [14]. In their analysis, they found that the use of any inhaled antibiotic was beneficial for the patients as it resulted in a marked decline in long-standing bronchial infection. There was a reduction in PA-associated infections from 81% to 52% and for other bacterial infections from 29.4% to 10%. Sputum production reduced from 31.6% to 19% and the presence of mucopurulent sputum fell from 29.4% to 10%. The Wilson et al. study focused on the role of DPI containing ciprofloxacin [21]. It reported a drastic reduction in colony-forming units in the treatment group, especially on the eighth day, after which the count gradually increased (day 29 onwards). The eradication rate was 48% in the ciprofloxacin group versus 12% in the placebo. On day 84, both groups had similar results. Among the secondary outcomes, reduced exacerbation, antibiotic use, and hospitalization were reported compared to a placebo. It is worthwhile to mention that improvements in clinical parameters were observed only in the early weeks of follow-up.

Respire 1, divided patients into two arms (14 days on/off and 28 days on/off) of cycles using ciprofloxacin as the DPI. It observed statistically more favorable outcomes when using the 14-day off-on regime (336 days to first exacerbation versus 186 days in placebo). It demonstrated that other parameters were also less pronounced in the 28-day regimen [19]. While respire 1 was done on patients from Europe, America, Australia, New Zealand, and Japan, the respire 2 study included patients from Africa and Asia in addition to the previously mentioned. It included 521 patients that were subdivided into 176 on 14 days and 171 on 28 days and the rest in the placebo category. However, no subgroup demonstrated superiority [17]. Exacerbation was not experienced in 61.4% and 67.3% of patients in the 14 and 28-day cycle categories, while for the placebo population it was 58%. The incidence rate of exacerbation was 17% and 45%, respectively, but neither reached the expected target. The orbit 2 trial, specifically designed for patients with PA infection, reported an increase in time to exacerbation (134 in treatment versus 58 days in placebo), coupled with a sharp decline in the bacterial density from the startup up to day 28 [20].

The British Thoracic Society recommends early and long-term use of oral antibiotics followed by nebulized or intravenous varieties in an attempt for the efficacious treatment of PA [22]. The successful elimination and prevention of infection is the main pillar in the management of such patients. However, despite the recent focus on NCFB, there is a current vacuum in comparative trials on the efficacy of inhaled antibiotics. Factors such as lack of support, gaps in knowledge of disease and management protocols, and non-receptive lung pathology continue to serve as a challenge for researchers. There is still no consensus on the duration of the antibiotic course, the preferred target age group of patients, or the high-quality guidelines for their use [23,24]. In addition, the true incidence of the emergence of drug-resistant organisms also remains unknown.

Limitations

This review only included articles that were available for free download and were published in English text. In addition, some articles that included the pediatric population were also excluded.

## Conclusions

This study focused on the comparison of tolerability, adverse effects, and effectiveness of different forms of inhaled antibiotics administered to patients with NCFB. It was observed that all forms of inhaled antibacterial benefit the patient when given in addition to the oral and intravenous varieties. The most favorable outcomes are the delay of exacerbation, decline in hospital admission, and lowering of the microbial count in the sputum. However, it is difficult to comment on the superiority among the different subtypes as comparison trials are limited. Also, the study population demographics, duration of antibiotics, and mode of administration are different. It can, however, be deduced that amikacin is a cheaper option and can be of value, especially in low economic zones. Our research also found colistin being generally better tolerated than tobramycin, evidenced by a lower incidence of cough and bronchospasm. Furthermore, the liposomal form of ciprofloxacin has fewer adverse effects when compared to the regular form. There is a strong need for more research to establish algorithms and guidelines for antibiotic therapy in such patients as exacerbations due to reinfections remain the main reason for multiple admissions. Comparative trials are the need of the hour as this would not only benefit the patients but also reduce the socio-economic burden on the health facilities as well.
